# Assessment of Neck Muscle Shear Modulus Normalization in Women with and without Chronic Neck Pain

**DOI:** 10.3390/diagnostics12081791

**Published:** 2022-07-23

**Authors:** Angela V. Dieterich, Utku Şükrü Yavuz, Frank Petzke, Antoine Nordez

**Affiliations:** 1 Physiotherapy, Faculty of Health, Security, Society, Furtwangen University, 78120 Furtwangen im Schwarzwald, Germany; 2 Biomedical Signals and Systems, University of Twente, 7522 NB Enschede, The Netherlands; s.u.yavuz@utwente.nl; 3 Pain Clinic, Department of Anesthesiology, University Medical Center, 37075 Göttingen, Germany; frank.petzke@med.uni-goettingen.de; 4 Movement-Interactions-Performance, Nantes Université, EA 4334, F-44000 Nantes, France; antoine.nordez@univ-nantes.fr; 5 Institut Universitaire de France (IUF), F-75000 Paris, France

**Keywords:** elasticity imaging techniques, muscle, neck pain, data processing

## Abstract

Identifying the objective stiffness of the neck muscles facilitates the early and specific diagnosis of neck pain and targeted therapy. However, individual variation in the muscle shear modulus obscures differences between healthy and diseased individuals. Normalization may improve the comparability between individuals. The shear modulus at different functional tasks served as a reference for normalizing the neck muscles’ shear modulus of 38 women, 20 with chronic neck pain and 18 asymptomatic. Reference tasks were *maximal voluntary contraction*, *relaxed sitting*, *prone head lift*, *balancing 1 kg on the head, and neck extension at 48 N*. The effects of normalization on within-group variation and between-group differences were compared. Normalization with *maximal voluntary contraction* was discarded due to imaging problems. Normalization with *relaxed sitting*, *prone head lift*, *balancing 1 kg*, and *neck extension at 48 N* reduced within-group variation, by 23.2%, 26.8%, 11.6%, and 33.6%, respectively. All four normalization approaches reduced the *p*-values when testing for between-group differences. For the pain group, normalization with *relaxed sitting* and *head lift* indicated less normalized muscle stiffness, while normalization with *balancing 1 kg* and *extension at 48 N* indicated higher stiffness. The contradictory results are explainable by non-significant group differences in the reference tasks. Normalization of the muscle shear modulus is effective to reduce within-group variation, but a trustworthy normalization approach for group comparisons has yet to be identified.

## 1. Introduction

Neck pain is among the most common causes of disability [1]. Although periods of acute pain are typically short [2], rates of recurrence and chronicity are high [3]. Perceived muscle tension increases the risk of developing neck pain [4] and contributes to pain symptoms [5,6]. Thus, objective measures of neck muscle elasticity may aid early diagnosis, identification of myofascial pain components, and targeted therapy. The objective elasticity or stiffness of the neck muscles can be examined using ultrasound shear wave elastography (SWE), which non-invasively, via shear wave speed, measures the muscle shear modulus in superficial and deep-lying muscles [7] during passive and active muscle states [8]. Thus, passive tissue properties and functional muscle performance can be evaluated [9,10,11]. However, a muscle’s typical shear modulus differs between individuals. Apart from pathology and active muscle tension, it is influenced by the individual’s constitution, age, and gender [12,13,14]. The individual variation of a muscle’s shear modulus impedes the comparability of measurements, the definition of typical stiffness, and, therewith, the detection of differences or deficiencies. Furthermore, measurements of the muscle shear modulus should not be compared between the systems of different SWE manufacturers [15].

Normalization, i.e., the expression of a measurement as a percentage of an individual reference value, may be used to improve the comparability of measurements between individuals [16]. Normalization aims to reduce measurement variation that arises from factors that are not targeted in an investigation [17,18]. For example, electromyography measurements of muscle activation must be normalized to enable comparisons between individuals, muscles, measurement occasions, and different studies. In electromyography, the most broadly used and recommended reference for normalization is from a maximal voluntary isometric contraction (MVIC) [19]. In individuals with pain, the exertion of maximal force may not be feasible [17,20]. Alternatively, tasks with submaximal effort have been advocated as a reference for normalization [21]. As far as we are aware, measurements of the muscle shear modulus have been normalized only in two studies in which the muscle shear modulus during relaxation has been used as the reference [7,22]. We hypothesized that normalization of the muscle shear modulus reduces within-group variation and facilitates the detection of a between-group difference.

In a recent study on the stiffness of the neck muscles of women with chronic neck pain and asymptomatic women, we found high within-group variation and an insignificant trend of lower neck muscle stiffness in the pain group [23]. Using these existing data, the here presented work aimed to evaluate different normalization approaches by comparing within-group variance and between-group difference between raw (non-normalized) measures and different normalization approaches.

## 2. Materials and Methods

### 2.1. Material

**Figure 1:**
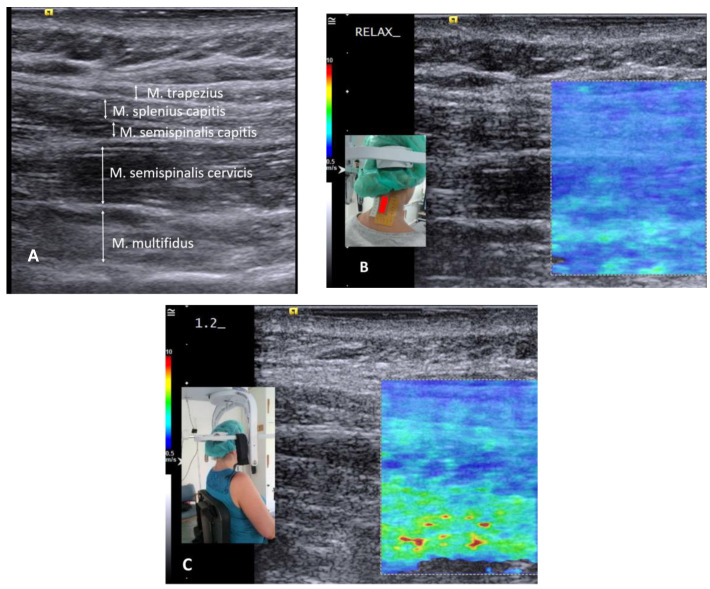
Example images of a control subject: (**A**) neck extensor muscles in ultrasound B-mode; elastogram of the neck muscles during (**B**) relaxed sitting (low stiffness in blue) and (**C**) isometric neck extension at 12 N (high stiffness in red). (**B**) includes a photograph with the transducer location (red) framed with adhesive foam to facilitate repeatability; (**C**) shows setup of the graded force task.

**Figure 2:**
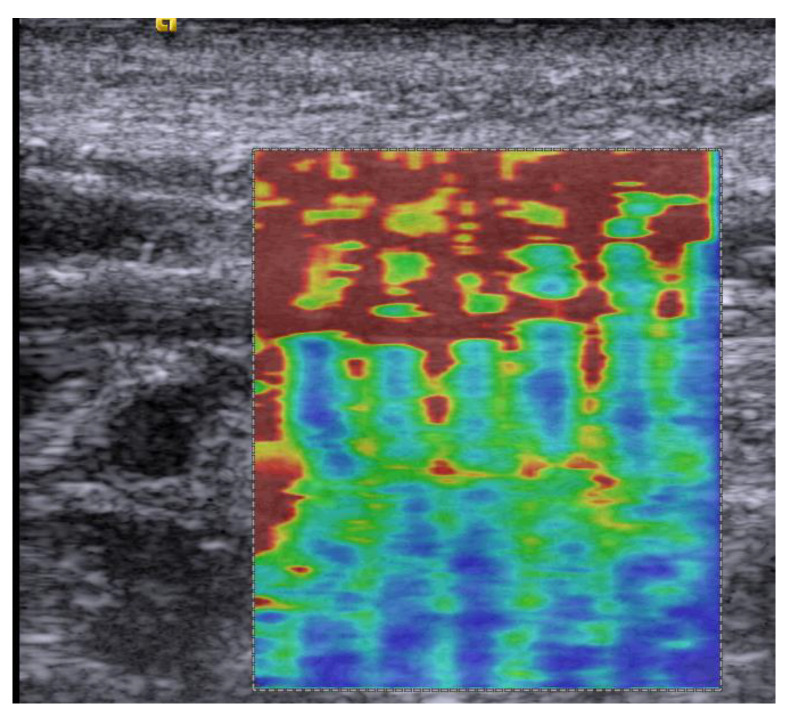
Elastogram of a maximal voluntary isometric contraction with a discontinuous, artifactual appearance and saturated measurements (dark red color).

Data were captured in an already published cross-sectional case-control study including 38 women (median age 50.5 years, IQR 9 years) [23]. Participants were recruited at a university pain clinic. There, the study took place in a laboratory. Twenty women with chronic neck pain >6 months and a score >10/50 on the Neck Disability Index (neck pain group), and 18 age-matched asymptomatic women without a history of recurrent lower back or neck pain (control group) performed multiple tasks, during which the shear modulus of the neck extensor muscles was measured using ultrasound SWE (Acuson S3000; Siemens, Erlangen, Germany) (Figure 1 and Figure 2). Exclusion criteria for both groups included systemic pathologies and medications that may influence muscle tone or performance, pregnancy, and BMI >30 kg/m^2^ (image quality). Prior to data collection, information on the expected group difference had not been available; the sample size was chosen in agreement with other relevant studies [24,25]. The study tasks of *graded isometric neck extension* and *MVIC* while sitting in a dynamometry-equipped measurement unit (MultiCervical Unit (MCU), BTE Technologies, CO, USA) (Figure 1C) served to observe the increase of neck muscle stiffness with force; a standardized *deskwork task* under time pressure meant to mimic stressful office work; in prone the *resting state* and *head lift* were recorded to monitor the individual ability to relax, or to lift the weight of the head; the task of *balancing sandbags of 1 kg* on the head was inspired by a clinically used exercise and deemed easy to standardize. All tasks were repeated three times. Muscle stiffness measured over all scanned neck muscles, including the trapezius, splenius capitis, semispinalis capitis, semispinalis cervicis, and multifidus muscles (Figure 1A) was recorded in longitudinal transducer orientation approximately 1 cm lateral to the spinous processes, centered at C4 (Figure 1B), at 9 MHz with a 9L4 linear transducer with 4 cm footprint. The thyroid preset and the maximal range of shear wave speed (10 m/s) was set. Gain (14–20 dB), dynamic range (45–65 dB), and image depth of 3.5–4.5 cm were adjusted for good visualization. The shear modulus was computer-extracted over the complete elastogram (all visible neck extensor muscles) and also in muscle-specific regions using a custom MATLAB program [23]. Stiffness measurements were averaged over the three task repetitions. The visual check of data quality demonstrated artifacts [26,27] in 28% of the elastograms of *MVIC*, and shear wave velocities exceeding 9.98 m/s, suggesting measurement saturation [26], in 30% of the *MVIC* trials (Figure 2). *MVIC* data were excluded. Measurements of the *resting state* demonstrated insufficient reliability, ICC_3,3_ = 0.138, and were excluded [23]. Data from nine tasks with active muscle performance remained in the analysis (Table A1 in Appendix A). The insufficient data quality of *MVIC* and *resting state* prevented their use as references for normalization.

Due to potential variation in force sharing between muscles of similar function [23,28], the robustness of the measures, and the complexity of comparisons, this report is limited to the shear modulus of the neck muscle group, as measured over the entire elastogram, without specifying muscle regions.

### 2.2. Normalization

**Figure 3:**
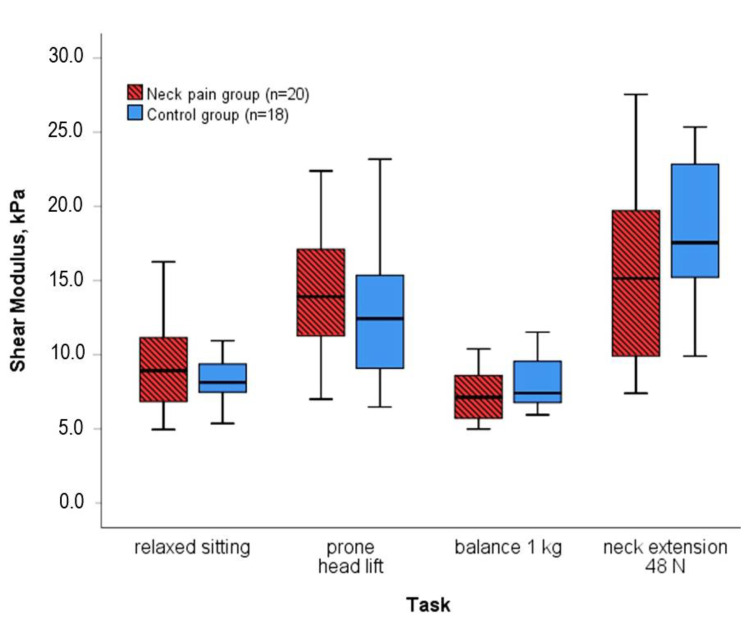
Neck muscle shear modulus of the four tasks that have been used as a reference for normalization. Note that median stiffness in the pain group tended to be higher, similar, or lower compared to the control group (not significant).

The individual shear modulus during four tasks of submaximal force requirements served as the reference value for normalization (Figure 3). Each measurement of the neck muscles’ shear modulus of an individual was normalized with each of the four individual reference values, i.e., 180 (20 participants with neck pain*9 tasks) and 162 (18 pain-free participants*9 tasks) measurements were transformed into a percentage of each of the four alternative reference values.

The reference tasks were chosen because they were common all-day activities of functional relevance: (a) *relaxed sitting* (instruction to sit relaxed in the MCU unit; participants were meant to reproduce their usual behavior) and (b) prone *head lift*. Alternatively, the tasks were deemed easy to standardize: (c) *balancing 1 kg* on the head and (d) isometric *neck extension at 48 N* (which was the highest resistance in the graded force setup of the first task). Notably, the performance between groups tended to differ in a task-dependent manner. Median stiffness in the pain group compared to the control group was higher during tasks (a) and (b), quasi-equal in (c), and lower in (d) (Figure 3).

### 2.3. Statistical Analysis

The mean of the raw and the normalized stiffness measures of each participant was computed, i.e., nine measurements of different activities were averaged per participant for comparing groups. The Shapiro–Wilk test indicated skewed data distribution in the raw measures of the neck pain group (*p =* 0.001) and with normalization to *head lift* in both groups (both *p* < 0.001). To enable the comparison of all measures, within-group variation was described using the ratio of the interquartile range (IQR) divided by the median as a non-parametric coefficient. The change of variation with each normalization approach was expressed as a percentage of the variation of the raw measures. Between-group difference was examined using the non-parametric Mann–Whitney test, and the parametric independent *t*-test for the measures with normal distribution. For the additional examination of single tasks, the Shapiro–Wilk test indicated skewed data distributions. The between-group difference of each task was examined using the Mann–Whitney test. Statistics were computed with the α-level at 0.05 (SPSS v.26, IBM, Ehningen, Germany).

## 3. Results

All four normalization approaches reduced within-group variation of the measures of neck muscle stiffness that had been averaged over tasks (Table 1). All four normalization approaches led to smaller *p*-values, suggesting improved detection of a between-group difference (Table 1). Notably, normalization with *relaxed sitting* and *head lift* replicated the finding of less neck muscle stiffness in the pain group, while normalization with *extension at 48 N* and *balancing 1 kg* indicated higher normalized stiffness in the pain group (Table 1). Muscle stiffness in the pain group compared to the control group during the *head lift* task was significantly lower when normalized to *extension at 48 N*, and significantly higher during the *extension at 48 N* task when normalized to *head lift*, both *p* = 0.017 (Table A1 in Appendix A).

## 4. Discussion

All four normalization approaches reduced within-group variation and facilitated the detection of a between-group difference. However, the group difference was not consistent between normalization approaches. In support of the trend in the raw measurements, two normalization approaches demonstrated less stiffness of the neck muscles in the neck pain group. The other two approaches indicated the opposite result.

Inconsistent results from different normalization procedures have been reported in electromyography (EMG) studies [29,30,31]. Recently, a normalization matrix has been published to provide advice on the advantages and disadvantages of different EMG normalization methods [17]. The authors recommended normalization with the MVIC if feasible, due to the unique advantage of interpreting normalized measures as the percentages of maximal force. Due to measurement saturation and artifacts, the MVIC measures in this study could not be used for normalization. To date, MVIC has not been used for normalizing SWE measures.

Submaximal efforts provide an alternative when an MVIC cannot be performed. However, as supported by the current results, a reference value that differs between groups may be problematic for comparing normalized measures between groups [17]. In the included reference tasks, the shear modulus did not differ significantly between groups but trends were visible. Larger trends of different medians in the reference tasks *head lift* and *extension at 48 N* (Figure 3) were associated with smaller *p*-values of the between-group difference in the normalized measures. In addition, the direction of the difference in the group medians of the reference tasks appears to have influenced the direction of the between-group difference in the normalized results, e.g., relative to the higher group median in *prone head lift*, normalized measures were lower in the pain group. Conclusively, the choice of the reference measure influences the result of a group comparison with normalized measures. The inconsistent results question all four of the here presented normalization approaches for the purpose of group comparisons.

Besomi et al. warned against using fixed force values for normalization in group comparisons if the effort differs between groups [17]. We recorded an MVIC of 56 N ±19 N in the pain group versus 64 N ± 19 N in the control group, *p* = 0.219 [23]. Thus, the effort for an *extension at 48 N* was higher in the pain group. Relative to the pain group’s low shear modulus during *extension at 48 N* (Figure 3) as normalization reference, the pain group’s relatively high muscle shear moduli during, for example, *head lift* transformed into high percentages in the normalized measures. This probably explains the opposite group difference with normalization to *extension at 48 N,* and likewise to *balancing 1 kg*.

In concordance with the recommendations for normalizing EMG measures of muscle activation, we used averaged measures from task repetitions and we did not normalize the measures of single muscle regions [17]. The distribution of muscle tension during a submaximal effort may vary between synergistic muscles, task repetitions, and groups [23,28,32]. Thus, an average over the group of synergistic muscles is more robust.

Measurements were taken along the main shortening axis of the neck extensor muscles, as validated [33,34], but in some of the muscles, the transducer was not aligned with muscle fiber direction. For example. the stiffness contribution of the oblique splenius capitis muscle may be affected by the deviating fiber angle, presumably to a small amount [34,35]. The influence of angle deviations of muscle fibers affected both groups similarly and may have reduced the precision of the cumulated neck extensor shear modulus, but not the comparison between groups or normalization approaches.

## 5. Conclusions

Normalization of the muscle shear modulus enhances the comparability between individuals by reducing within-group variation. The detection of a between-group difference is facilitated. From this perspective, the normalization of measurements of the muscle shear modulus is promising. On the other hand, normalization with functional tasks performed differently between groups results in questionable inferences. Further normalization approaches, e.g., a defined percentage of the maximal force, must be tested to identify a trustworthy normalization approach for measurements of the muscle shear modulus.

## Figures and Tables

**Table 1:** Raw and normalized neck muscle shear modulus averaged over tasks per group, coefficients of within-group variation, change of variation relative to the raw measurements, the main result of the group comparison, and *p*-values for differences between groups. Abbr.: IQR, interquartile range; n.a. not applicable; * significant at *p* < 0.05.

	Raw Shear Modulus, kPa		Shear Modulus % of Relaxed Sitting		Shear Modulus % of Head Lift		Shear Modulus % of Balancing 1 kg		Shear Modulus % of Extension at 48 N	
	Neck Pain	Control	Neck Pain	Control	Neck Pain	Control	Neck Pain	Control	NeckPain	Control
Shear modulus median (IQR)	13.1 (6.4)	13.6 (5.6)	139.6 (58.5)	155.9 (43.5)	94.2 (34.4)	107.8 (31.8)	188.9 (83.0)	174.4 (62.2)	89.1 (30.7)	76.5 (19.6)
Coefficient IQR/median	0.49	0.41	0.42	0.28	0.37	0.29	0.44	0.36	0.34	0.26
Result for the neck pain group	3.3% lower stiffness		10.5% lower normalized stiffness		12.6% lower normalized stiffness		8.3% higher normalized stiffness		16.6% higher normalized stiffness	
Mann-Whitney *p*-value	0.654		0.317		0.059		0.251		0.082	
T-Test *p*-value (if applicable)	n.a.		0.242		n.a.		0.362		0.035 *	

## Data Availability

The data presented in this study are openly available on zenodo.org, https://doi.org/10.5281/zenodo.6638939.

## Appendix A

**Table A1:** Raw and normalized neck muscle shear modulus (median (interquartile range (IQR))) per task; *p*-value of the Mann–Whitney test for the between-group difference (in italics). * significant at *p* < 0.05 (in bold).

	Raw Shear Modulus, kPa		Shear Modulus % of Relaxed Sitting		Shear Modulus % of Head Lift		Shear Modulus % of Balancing 1 kg		Shear Modulus % of Extension at 48 N	
	Neck Pain	Control	Neck Pain	Control	Neck Pain	Control	Neck Pain	Control	Neck Pain	Control
Relaxed sitting	8.9 (4.6)	8.1 (2.3)			65 (25)	69 (33)	121 (68)	102 (38)	58 (40)	48 (13)
*p*-value	*0.740*				*0.593*		*0.093*		*0.149*	
Extension at 12 N	9.3 (8.5)	10.7 (5.7)	115 (48)	124 (37)	74 (45)	80 (29)	145 (86)	122 (71)	74 (35)	63 (25)
*p*-value	*0.573*		*0.426*		*0.290*		*0.496*		*0.099*	
Extension at 24 N	10.8 (8.2)	13.7 (9.9)	134 (57)	162 (90)	86 (41)	95 (38)	171 (96)	168 (102)	81 (21)	75 (14)
*p*-value	*0.317*		*0.196*		*0.093*		*0.828*		*0.217*	
Extension at 36 N	14.8 (12.0)	14.7 (8.1)	157 (96)	182 (85)	109 (69)	123 (77)	195 (150)	198 (90)	96 (36)	90 (13)
*p*-value	*0.496*		*0.377*		*0.149*		*0.740*		*0.158*	
Extension at 48 N	15.1 (9.9)	17.6 (8.5)	173 (109)	207 (56)	**108** **(48)**	**141** **(63)**	225 (169)	218 (120)		
*p*-value	*0.149*		*0.149*		***0.017** **		*0.919*			
30° Rotation at 24 N	12.7 (7.8)	15.6 (9.0)	143 (73)	186 (63)	94 (52)	120 (57)	204 (138)	169 (96)	95 (34)	87 (34)
*p*-value	*0.377*		*0.167*		*0.093*		*0.534*		*0.696*	
Office stress	16.6 (11.1)	16.3 (10.4)	187 (95)	194 (85)	129 (72)	138 (69)	234 (154)	233 (119)	117 (92)	92 (42)
*p*-value	*0.998*		*0.478*		*0.317*		*0.496*		*0.099*	
Balancing 1 kg	7.1 (3.2)	7.4 (3.3)	83 (39)	98 (35)	53 (35)	74 (41)			45 (43)	46 (29)
*p*-value	*0.141*		*0.093*		*0.105*				*0.919*	
Prone head lift	13.9 (6.3)	12.4 (6.5)	153 (59)	145 (71)			189 (124)	136 (105)	**93** **(46)**	**71** **(32)**
*p*-value	*0.393*		*0.593*				*0.105*		***0.017** **	
